# Symmetry warrants rational cooperation by co-action in Social Dilemmas

**DOI:** 10.1038/srep13071

**Published:** 2015-08-12

**Authors:** V. Sasidevan, Sitabhra Sinha

**Affiliations:** 1 The Institute of Mathematical Sciences, CIT Campus, Taramani, Chennai 600113, India

## Abstract

Is it rational for selfish individuals to cooperate? The conventional answer based on analysis of games such as the Prisoners Dilemma (PD) is that it is not, even though mutual cooperation results in a better outcome for all. This incompatibility between individual rationality and collective benefit lies at the heart of questions about the evolution of cooperation, as illustrated by PD and similar games. Here, we argue that this apparent incompatibility is due to an inconsistency in the standard Nash framework for analyzing non-cooperative games and propose a new paradigm, that of the co-action equilibrium. As in the Nash solution, agents know that others are just as rational as them and taking this into account lead them to realize that others will independently adopt the same strategy, in contrast to the idea of unilateral deviation central to Nash equilibrium thinking. Co-action equilibrium results in better collective outcomes for games representing social dilemmas, with relatively “nicer” strategies being chosen by rational selfish individuals. In particular, the dilemma of PD gets resolved within this framework, suggesting that cooperation can evolve in nature as the rational outcome even for selfish agents, without having to take recourse to additional mechanisms for promoting it.

Strategic interactions occur all around us in a multitude of forms between autonomous *agents*. These interacting agents could correspond to individual humans or animals or even computer algorithms, as well as, collective entities such as groups, organizations or nations. Analyzing their interactions in terms of games^1^ is a promising approach for understanding the behavior of a wide variety of socio-economic and biological systems, and finds applications in fields ranging from economics and political science to computer science and evolutionary biology^2^. A *game* is described by the set of all possible actions by a specified number of agents, where each possible combination of actions is associated with a payoff for each agent. Thus, the payoff received by an agent depends on her choice of action, as well as that of others. Agents are assumed to be rational and selfish, who want to maximize their individual payoffs. In addition, every agent knows that all agents satisfy these criteria (for a detailed discussion of these ideas see, e.g., Ref. 3). Each of these assumptions is crucial in determining the outcome of a game. While they may or may not hold in specific real-life scenarios, the agent behavior embodied by these assumptions provides a crucial benchmark for strategic behavior.

In order to solve a game, i.e., to find the set of actions that the agents will employ given the structure of the game, one needs a solution concept that will form the basis for strategy selection by the agents. For non-cooperative games, where agents choose their actions independently without communicating with other agents, the canonical solution concept employed is that of the Nash equilibrium. It is defined informally as the set of actions chosen by the agents where no agent can gain by unilaterally deviating from this equilibrium^4^. Nash equilibria exist for all games having a finite number of agents choosing from a finite set of actions, making it a very general concept that has wide applicability^5^. Indeed, the concept has been central to various attempts at developing quantitative descriptions of socio-economic phenomena^6^. However, analyzing specific games using the concept of Nash equilibrium can raise the following issues: (i) A game may have more than one Nash equilibria and hence, deciding which of these will be adopted by rational agents is a non-trivial problem^7^. Additional criteria need to be provided for selecting an equilibrium; however their success is not always guaranteed^8^. (ii) The Nash equilibrium of a game may sometimes be inferior to an alternative choice of actions by the agents in which all the parties get higher payoff. This gives rise to apparently paradoxical situations in games representative of social dilemmas, such as the Prisoner’s Dilemma (PD)^9^, the Traveler’s Dilemma^10^,^11^, etc. For example, in PD, where each agent has the option to either cooperate with the other agent or defect, mutual defection is the only Nash equilibrium, although mutual cooperation will result in higher payoffs for all agents. Results of experimental realizations of such games also show deviation from the Nash solutions^9^,^12^. That rational action by individual agents can result in an undesirable collective outcome for the agents is a long-standing puzzle^13^. In particular, it raises questions about how cooperation could have evolved and is maintained in natural populations^14^.

Here we argue that the genesis of this problem can be traced to a mutual inconsistency between the assumptions underlying the Nash equilibrium for symmetric game situations. One of these assumptions is that each agent is equally capable of analyzing the game situation and that all of them are aware of this. However, it is also assumed that agents can make *unilateral* deviations in their strategy, which is used to obtain a dominant strategy in games like PD. In other words, each agent looks *only* at the payoff structure of the game and takes a decision that is independent of *how* other agents decide. This is inconsistent with the earlier assumption because if the agents are aware that the others are also rational, they should take this (rational decision-making by the other agents) into account. To put it informally, the player will argue that “if the other player is like me, then she will be independently choosing the same strategy (although not necessarily the same action if it is a mixed strategy) as I, because we are faced with the same situation.” In this paper we present a novel solution paradigm for payoff-symmetric games, referred to as *co-action equilibrium*, that resolves this inconsistency, building on a concept originally introduced in the context of minority games^15^. As we shall see, the optimal action of rational agents in co-action equilibrium is markedly different from Nash equilibrium and leads to better collective outcomes, solving various social dilemmas such as PD.

The mutual inconsistency between (i) the assumption of players being aware that all of them are rational and (ii) the possibility of a dominant strategy, had been earlier pointed out informally in the specific context of PD—although, to the best of our knowledge, there have been no attempts to develop a quantitative framework that addresses this problem. In what is possibly the earliest statement about the rationality of cooperation in PD, Rapoport^16^ had argued that because of the symmetry of the game, rational players will choose the same action—and as it involves a higher payoff, they will always opt for mutual cooperation. This argument has been independently put forward by Hofstadter^17^ in the context of a *N*-person PD. The response of conventional game theory to this line of reasoning, as set forth at length by Binmore^18^, centers on the argument that these approaches crucially rely on constraining the set of feasible outcomes of the game to the main diagonal of the payoff matrix, thereby making it effectively a collective decision-making process^19^. As outlined in detail below, the co-action approach presented here allows the agents access to the full set of outcomes in the game matrix and the solution is obtained without restricting their choices of action. It is also general, applying to all symmetric non-cooperative games. As the theory of strategic interactions is central to the analysis of many phenomena across economics, social sciences and evolutionary biology, the co-action concept could potentially lead to new insights across a broad range of disciplines.

In this paper we analyze single-stage games with two actions per agent, where the payoff structure is unchanged on exchanging the identities of the agents (payoff symmetry). We primarily focus on two-person games, with agents playing the game once (in contrast to repeated games where agents can interact many times in an iterative manner) and analyze in detail three well-known instances, viz., PD, Chicken (also referred to as snow-drift or Hawk-Dove) and Stag Hunt. These games model a wide variety of conflict situations in nature where cooperation may emerge under certain circumstances^20^. We describe the co-action solution for these games which, in general, leads to “nicer” strategies being selected by the agents compared to the Nash solution. For example, the co-action equilibrium in PD corresponds to full cooperation among agents at lower values of temptation to defect, while for higher temptation each agent employs a probabilistic strategy. Thus, co-action typically results in more globally efficient outcomes, reconciling the apparent conflict between individual rationality and collective benefit. Further, the co-action equilibrium is unique and therefore, agents are not faced with the problem of equilibrium selection. The concept can be extended to other scenarios, such as, symmetric games involving several players, or even non-symmetric games when agents can be grouped into clusters with symmetry holding within each. In fact, the latter case can be seen as defining a new class of games between players, where each “player” represents a group of agents who independently choose the same strategy.

**The Co-action equilibrium**. To describe the co-action solution concept, we consider the general case of a payoff-symmetric, two-person game where each agent (say, *A* and *B*) has two possible actions (Action 1 and Action 2) available to her. Each agent receives a payoff corresponding to the pair of choices made by them. If both agents choose the same option, Action 1 (or 2), each receives the payoff *R* (or *P*, respectively), while if they opt for different choices, the agent choosing Action 1 receives payoff *S* while the other receives *T*. Thus, the game can be represented by a payoff matrix that specifies all possible outcomes (Fig. 1). An agent may employ a mixed strategy, in which she randomly selects her options, choosing Action 1 with some probability *p* (say) and Action 2 with probability (1 − *p*). A pure strategy corresponds to *p* being either 0 or 1. A Nash equilibrium for a game can be in pure strategies or in mixed strategies. As noted earlier, a given game may have more than one Nash equilibrium, possibly involving mixed strategies. Assuming that agent *A* ( *B*) chooses Action 1 with probability *p*_1_ (*p*_2_) and Action 2 with probability 1 − *p*_1_ (1 − *p*_2_, respectively), their expected payoffs are,


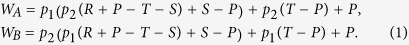


The symmetry of the game is reflected in the fact that *W*_*A*_ and *W*_*B*_ are interchanged on exchanging *p*_1_ with *p*_2_. It is easily seen that if a mixed strategy Nash equilibrium exists, it is the same for both agents and given by the probabilities


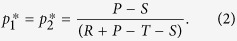


The Nash solution assumes that all agents are rational and that each agent knows the planned equilibrium strategies of the other agents. Furthermore, a unilateral deviation in strategy by one of them will not change the strategy choice of others (who are assumed to be just as rational as the one who deviated!). This is implicit in Eq. (1) where each agent maximizes her payoff independent of the strategy of the other agent. In other words, while making a choice the agents do not take into account the fact that the other agents (who are assumed to have identical capabilities) are also deciding simultaneously on their choice and that they are all aware of this. Although this latter assumption is deeply embedded in standard game theory, it is inconsistent with the assumption that every agent is aware that all other agents are just as rational as them. By contrast, in the co-action concept, by virtue of the symmetry of the game, each agent will argue that whatever complicated processes she employs in arriving at the optimal decision, the other agents will choose the same strategy as they have the same information and capabilities. It is important to note that this does not require any communication between the agents nor does it invoke the existence of trust or other extraneous concepts. Rather, it arises from the fact that both agents are equally rational and being in a symmetric situation, will reach the same conclusion about the choice of strategy; moreover, *they realize and consider this in making their decision*. It is important to note that the co-action concept does not imply that both agents will necessarily end up choosing the same action. For instance, the co-action solution for the single-stage PD is not to always cooperate—which distinguishes the present approach from the earlier arguments of Rapoport^16^ and Hofstadter^17^ where all agents always choose the same action—but to resort to a mixed strategy when the temptation to defect is sufficiently high.

In the co-action concept, each agent maximizes her payoff assuming that all other agents in a symmetric situation will be making the same decision. Formally this amounts to optimizing the expected payoff functions of each of the two agents, which in this case are identical:


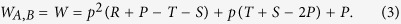


Here *p* is the probability with which each of the agents *A* and *B* chooses Action 1. Under the co-action concept, the equilibrium strategy p* of the agents is obtained by maximizing *W* with respect to 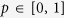. If the maximum of function *W* in [0, 1] occurs at one of the ends (i.e., *p* = 0 or 1), it results in a pure strategy co-action equilibrium. However, if *W* has a maximum inside (0, 1) then the co-action equilibrium is a non-trivial mixed strategy, viz.,


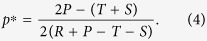


The existence of the co-action equilibrium for all symmetric games is guaranteed from the smoothness of polynomial functions such as Eq. (3). Also, unlike the Nash equilibrium, the co-action equilibrium is unique and thus, for a given symmetric game there is no ambiguity about the optimal choice of action for the agents.

**Case studies**. Having described the concept of co-action equilibrium, we will now apply it to three well-known two-person symmetric games, illustrating in each case the differences between the co-action and Nash equilibria. Each of these games is defined in terms of a specific hierarchical relationship between the payoffs *R*, *S*, *T* and *P* (using the terminology of the payoff matrix shown in Fig. 1).

**Prisoner’s Dilemma**. PD is one of the most well-studied games in the literature of strategic choices in social sciences and evolutionary biology^21^,^22^. It is the canonical paradigm for analyzing the problems associated with evolution of cooperation among selfish individuals^14^. The game represents a strategic interaction between two agents who have to choose between cooperation (Action 1) and defection (Action 2). If both players decide to cooperate, each receives a “reward” payoff *R* and if both players decide to defect, then each receives a “punishment” payoff *P*. If one of the players decides to defect and the other to cooperate, then the former gets a payoff *T* (often termed as the “temptation” to defect) and the latter gets the “sucker’s payoff” *S*.

In PD the hierarchical relation between the different payoffs is *T* > *R* > *P* > *S*. The only Nash equilibrium for this game is both agents choose defection (each receiving payoff *P*), as unilateral deviation by an agent would yield a lower payoff (*S*) for her. Note that, mutual defection is the only Nash solution even if the game is repeatedly played between the players a finite number of times. However, it is easy to see that mutual cooperation would have resulted in a higher payoff (*R*) for both agents. This illustrates the apparently paradoxical aspect of the Nash solution for PD where pursuit of self-interest by rational agents leads to a less preferable outcome for all parties involved. The failure on the part of the agents—who have been referred to as “rational fools”^23^—to see the obviously better strategy is at the core of the dilemma and has important implications for the social sciences, including economists’ assumptions about the efficiency of markets^24^. Further, experimental realizations of PD show that some degree of cooperation is achieved when the game is played by human subjects, which is at variance with the Nash solution^9^,^12^,^25^.

In more general terms, PD raises questions about how cooperation can emerge in a society of rational individuals pursuing their self-interest^14^ and there have been several proposals to address this issue. These have mostly been in the context of the iterative PD (rather than the single-stage game that we are considering here) and typically involve going beyond the standard structure of the game, e.g., by introducing behavioral rules such as direct or indirect reciprocity^26^, assuming informational asymmetry^27^, etc. By contrast, in the co-action solution, rational selfish agents achieve non-zero levels of cooperation in the standard single-stage PD, with the degree of cooperation depending on the ratio of temptation *T* to reward *R*.

To obtain the co-action solution of PD, we use the formalism described earlier with the value of the lowest payoff *S* assumed to be zero without loss of generality. From Eq. (3) and using the hierarchical relation among the payoffs *T*, *R* and *P* for PD, it follows that when *T* ≤ 2*R*, the optimal strategy for the agents is p* = 1, i.e., both agents always cooperate. On the other hand, when the temptation to defect *T* > 2*R*, the optimal strategy is a mixed one with the probability of cooperation [Eq. (4)],


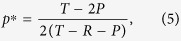


i.e., the agents randomly choose between the available actions, defecting with probability 1 − p*. As temptation keeps increasing, the probability of cooperation decreases and in the limit *T* → ∞, p* → 1/2, i.e., the agents choose to cooperate or defect with equal probability, receiving an expected payoff W* → *T*/4. Thus, unlike the Nash solution of PD where cooperation is not possible, the co-action solution of the game always allows a non-zero level of cooperation, with 1/2 < p* ≤ 1 [Fig. 2(a)]. The co-action solution also differs from the result expected based on the reasoning given by Rapoport and Hofstadter^16^,^17^—essentially a collective rationality argument—which suggests that rational agents will always cooperate. To the best of our knowledge, co-action is the first solution concept which allows probabilistic cooperation by the players in the single-stage PD.

The existence of non-zero level of cooperation in the co-action solution means that there is no longer any incompatibility between the individual actions of rational agents trying to maximize their payoffs and achieving the best possible collective outcome, thereby resolving the “dilemma” in PD. The co-action concept may be used to solve other games involving similar dilemmas such as traveler’s dilemma^10^. It is of interest to note in this context that in the various experimental realizations of PD, the level of cooperation observed is neither zero (as in the Nash solution) nor complete—the average being about 50% but with significant variation across experiments^25^. While it is unclear if such realistic game conditions conform to the idealized assumption of rational agents, the co-action solution does provide a benchmark strategy for these situations.

**Chicken**. Chicken (also referred to as Snowdrift or Hawk-Dove) is a two-person game that has been extensively investigated in the context of the study of social interactions and evolutionary biology^21^,^28^. It represents a strategic interaction between two agents who have to choose between being docile (Action 1) or being aggressive (Action 2). If both agents decide to be docile, they receive the payoff *R*, while if one is docile when the other resorts to aggression, the former—considered the “loser”—receives a lower payoff *S* (<*R*) and the latter—the “winner”—receives a higher payoff *T* (>*R*). However, the worst possible outcome corresponds to when both players choose to be aggressive, presumably resulting in severe damage to both, which is associated with the lowest payoff *P*. Thus, the hierarchical relation between the different payoffs in Chicken is *T* > *R* > *S* > *P*. Note that it differs from PD in that the payoff *S* is higher than *P*. Therefore, an agent benefits by being aggressive only if the other is docile but is better off being docile otherwise, as the cost of mutual aggression is high.

The game has three Nash equilibria, of which two correspond to pure strategies where one agent is docile while the other is aggressive. The mixed strategy Nash equilibrium 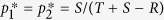 is given by Eq. (2), where it is assumed that the lowest of the possible payoffs *P* is zero [see Fig. 2(b)]. As in many other non-cooperative games with multiple Nash equilibria, one has to invoke additional criteria (viz., equilibrium refinements^7^) to decide which of these solutions will be selected by the agents. In Chicken, a commonly used refinement concept is that of evolutionarily stable strategy (ESS)^28^—an important concept in evolutionary game theory^29^—which, in this game, gives the mixed strategy Nash equilibrium as the unique solution.

To obtain the co-action solution for Chicken, we note that under this solution concept, agents choose their actions so as to optimize the payoff function Eq. (3). Using the hierarchical relation of the payoffs for Chicken (assuming the lowest payoff *P* is zero without loss of generality), it is easy to see that for 2*R* ≥ *T* + *S*, p* = 1 is the optimal choice. On the other hand, when 2*R* < *T* + S, agents choose to be docile with a probability [Eq. (4)],


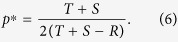


Thus, for low values of *T*, both agents decide to be docile (non-aggressive) always and avoid damaging each other, whereas, when the stakes are high (for large *T*) they randomly choose between the available actions, being docile with probability p* and aggressive with probability 1 − p*. As in PD, in the limit of large *T*, i.e., *T* → ∞, the optimal strategy is p* → 1/2, where the agents choose to be aggressive or docile with equal probability, receiving an expected payoff W* → *T*/4.

It is instructive to compare the optimal strategy of the agents under the different solution concepts when the stakes are very high. In the limit of *T* → ∞, the ESS suggests that both agents should resort to mutual aggression [i.e., 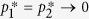 which is evident from Eq. (2)]. This would result in both agents suffering serious damage and receiving the lowest possible payoff *P*. Compared to this, the co-action concept yields a a significantly better outcome for both agents, as noted above. This difference is remarkable as the co-action solution shows that “nice” behavior among rational agents can occur even in a highly competitive environment. As in PD, experimental realizations of the single-stage game have reported a significant level (about 50%) of cooperative behavior^30^.

**Stag Hunt**. The last of the two-person games we discuss here is the Stag Hunt which is used to describe many social situations where cooperation is required to achieve the best possible outcome^31^. The game represents a strategic interaction between two agents who have to choose between a high-risk strategy having potentially large reward, viz., hunting for stag (Action 1) or a relatively low-risk, but poor-yield, strategy, viz., hunting for hare (Action 2). The agents can catch a stag (which is worth more than a hare) only if they both opt for it, i.e., cooperate, thereby receiving the highest payoff *R*. However, being unsure of what the other will do, they may both choose the safer option of hunting hare, which can be done alone, so that each receives a lower payoff *P*. However, if one agent chooses to hunt stag while the other decides to hunt hare, the former being unsuccessful in the hunt receives the lowest possible payoff *S*, while the latter (who succeeds in catching hare) gets the payoff *T*. Thus, the hierarchical relation between the payoffs in Stag Hunt is *R* > *T* ≥ *P* > *S*.

As in Chicken, the game has three Nash equilibria, of which two correspond to pure strategies where both agents opt for hunting stag or both choose to hunt hare. Note that both strategies are also evolutionarily stable, so that the ESS refinement, unlike in Chicken, does not yield a unique solution for this game. The mixed strategy Nash equilibrium 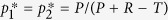 is given by Eq. (2) where it is assumed that the lowest of the possible payoffs *S* is zero.

The co-action solution for Stag Hunt is obtained by noting that as *R* is greater than *T*, the payoff function [Eq. (3)] increases monotonically in the interval [0, 1]. Thus, the co-action payoff *W* = *p*^2^(*R* + *p* − *T*) + *p*(*T* − 2*P*) + *P* is optimized when P* = 1, regardless of the values of *R*, *T* and *P*. Therefore, the solution of the game under the co-action concept is unique, with both agents opting to hunt stag, resulting in the best outcome for them. It may be of interest to note that experiments in single-stage Stag Hunt have reported that players tend to choose to coordinate on the higher payoff outcome in the majority of cases^31^,^32^,^33^. Unlike in the previous two case-studies, there is no conflict of interest among the agents playing Stag Hunt, who are instead trying to coordinate their actions in the absence of any communication. Thus, it can be viewed as a problem of equilibrium selection, with the co-action solution corresponding to the better one.

## Discussion

In this paper we have shown that the conflict between pursuit of individual self-interest and occurrence of collective outcomes that are mutually beneficial in the context of social dilemmas such as PD may only be an apparent one. The co-action concept presented here resolves this conflict by making mutually consistent assumptions about the behavior of rational agents. The different games that are analyzed in detail here show that the co-action solution concept leads to strategies that are relatively “nicer” and globally more efficient compared to the standard Nash equilibrium concept. In particular, it resolves the dilemma in PD as the mutually beneficial action, viz., cooperation, always has a significant probability (≥1/2) of being chosen by both agents. Similarly, co-action yields more cooperative outcomes in the other games, i.e., agents playing Chicken resort to non-aggressive strategies and agents achieve perfect coordination to receive the highest possible payoff in Stag Hunt. Thus, this solution concept reconciles the idea of individual self-interest pursued by rational agents with the achievement of collective outcomes that are mutually beneficial, even for single-stage games. While we do not claim that co-action is the only mechanism by which cooperation may originate and be maintained in nature, it certainly shows that cooperation can evolve among selfish rational agents. Note that our results do not depend on the specific definition of rationality one uses, as long as the same definition applies to all agents.

For an *N*-player (*N* > 2) game, if it can be considered as the set of all pair-wise interactions between agents who are symmetric in every respect, it is easy to see that the optimal co-action strategy will be exactly the same as that of the two-person game. The co-action solution concept can be generalized even to cases where the symmetry assumption does not hold across all agents. If the agents are aware that some of the other agents are different from them, one can still apply co-action within each cluster of agents (group) whose members consider each other to be identical (i.e., the symmetry assumption holds). For agents belonging to different groups, however, the payoffs are not invariant under interchanging the identities of the players. Thus, the symmetry of agents is broken across groups. For a population of agents whose members can be considered as belonging to two groups, one can treat the game as a two-player Nash-like scenario where each “player” is now a group of agents. However, unlike the standard Nash setting where one cannot have a mixed strategy as a stable Nash equilibrium, it is now possible for mixed strategy equilibria to be stable^34^. In general, one can consider a game with *N* agents, clustered into *M* symmetry groups, who have to choose between two actions. Assuming that the size of each group *i* is 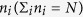, the payoff for an agent belonging to the *i*-th group is a polynomial of degree *n*_*i*_ in 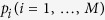, where each *p*_*i*_ is the probability of agents in that group to choose one of the actions. By contrast, the corresponding formulation of the game in terms of Nash solution concept will involve *N* variables with the payoffs being linear in each of these variables. Therefore, this defines a novel class of games between multiple clusters of agents, with agents independently choosing the same strategy as the other members of the cluster they belong to. The co-action results for such games may have potential implications for multi-agent strategic interactions, as in the tragedy of commons^35^.

While the results discussed here are in the context of idealized situations involving rational selfish agents, one may ask under what conditions would the co-action framework apply in real life. As we have outlined above, symmetry is a crucial ingredient for co-action thinking to apply. Such symmetry is more likely to be realized among members of a given community who share the same beliefs and a common identity. It has indeed been observed that cooperation is more common within an in-group than between agents belonging to different groups^36^. The significant levels of cooperative behavior reported in experimental realizations of social dilemmas (e.g., see Refs 9,25 for PD and Ref. 37 for its *N*-person generalization, i.e., the Public Goods game, Ref. 30 for Chicken and Refs 31, 32, 33 for Stag Hunt) could, to some extent, be explained by players ascribing to other players the same reasoning process as themselves and therefore resorting to co-action-like thinking. Experiments with human subjects playing PD have shown that the level of cooperation depends on the actual values of payoffs and in general decreases with the ratio of temptation for defection to reward for cooperation^25^–in line with the co-action solution. Also, players are known to employ non-deterministic strategies in PD realizations^38^, similar to what agents do in the co-action equilibrium for sufficiently high temptation. Game situations that allow “cheap talk” (i.e., communication between agents that does not directly affect payoff)^39^ which presumably allow players to affirm shared set of values—and thereby promote co-action thinking—have been shown to increase the level of cooperation in experiments^40^. In other experimental realizations, where players in a public goods game indicated their preferred contributions for different average levels of contribution by other group members, about half the players were observed to match what the others would do^41^. Such “conditional cooperation” could be an illustration of the symmetry considerations that players might engage in. Also, the co-action framework could provide a natural setting for the emergence of tag-based cooperation schemes among “sufficiently similar” agents^42^. The idea of “social projection”^43^ provides yet another instance where such considerations may be relevant. We note that there have been other approaches towards explaining cooperation in social dilemmas based on symmetry of the game situation, e.g., a recent model in which agents decide their strategies based on their most optimistic forecast about how the game would be played if they formed coalitions^44^.

In this paper we have focused on single-stage games but the co-action concept discussed here applies also to repeated games where information about the choices made by agents in the past are used to decide their future action. In this situation, the co-action solution developed in the context of single-stage games is applied at each iteration, with the past actions of agents used to define the different symmetry groups^15^. This is inherently a dynamical process, as the membership of these groups can evolve in time. For example, in iterative PD with *N* agents having memory of the choices made in the previous iteration, all agents who made the same decision in the last round will belong to the same symmetry group and will behave identically. The resulting solution can allow coexistence of cooperators and defectors in the game, which we will discuss in a future publication.

To conclude, we have introduced here a solution framework for non-cooperative games that resolves the apparent conflict between rationality of individual agents and globally efficient outcomes. It suggests that cooperation can evolve in nature as the rational outcome even with selfish agents, without having to take recourse to additional mechanisms for promoting it. In practice, the co-action and Nash solutions could represent two extreme benchmark strategies for non-cooperative games, the latter applying when the agents cannot be considered to be “sufficiently similar”. While we do not address here the question of which concept is more appropriate for a given situation, it is conceivable that agent behavior in reality may be described by a strategy between these two extremes and can potentially be represented by a combination of them. Although we have discussed co-action in the context of the evolution of cooperation among rational agents, the concept is far more general and could provide a mechanism for understanding strategic interactions across groups of sufficiently similar agents in many different settings.

## Additional Information

**How to cite this article**: Sasidevan, V. and Sinha, S. Symmetry warrants rational cooperation by co-action in Social Dilemmas. *Sci. Rep.* **5**, 13071; doi: 10.1038/srep13071 (2015).

## Figures and Tables

**Figure 1:**
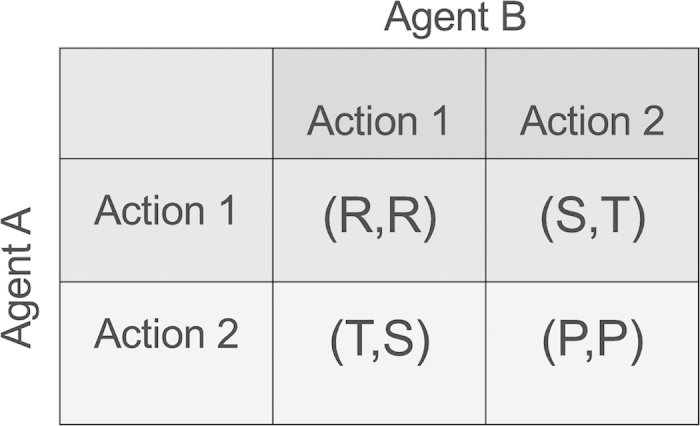
A generic representation of the payoff matrix for a two-person symmetric game where each agent has two actions available to her. For each pair of actions, the first entry in each payoff pair belongs to Agent *A* while the second belongs to Agent *B*. Different games discussed in the text, such as PD, Chicken and Stag-hunt, are defined in terms of different hierarchical relations among the elements *T*, *R*, *P* and *S*.

**Figure 2:**
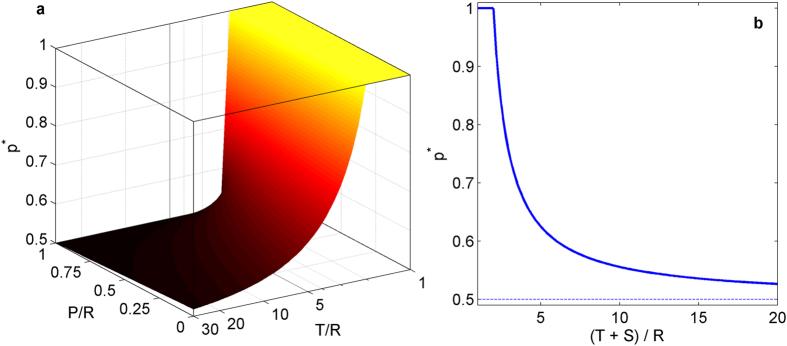
The variation of the optimal strategy—probability of choosing Action 1, P*—under the co-action solution concept for the games (**a**) Prisoner’s Dilemma (PD) and (**b**) Chicken, as a function of the payoff matrix elements *T*, *P*, *R* and *S*. In both games, for low values of *T* (corresponding to temptation for defection in PD and for being aggressive in Chicken), the agents always opt for Action 1 (corresponding to cooperation in PD and being docile in Chicken). However, as *T* increases, agents opt for a mixed strategy, where Action 1 is chosen with decreasing probability. In both cases, in the limit of very high *T*, the agent strategy becomes fully random with the two actions being chosen with equal probability. Note that in PD, the optimal strategy also has a very weak dependence on *P* (corresponding to punishment payoff for mutual defection).
